# Insights into the endophytic bacterial community comparison and their potential role in the dimorphic seeds of halophyte *Suaeda glauca*

**DOI:** 10.1186/s12866-021-02206-1

**Published:** 2021-05-12

**Authors:** Hongfei Wang, Manik Prabhu Narsing Rao, Yanli Gao, Xinyang Li, Rui Gao, Yuanguo Xie, Qiuli Li, Wenjun Li

**Affiliations:** 1 The Key Laboratory of Plant Biotechnology of Liaoning Province, School of Life Science, Liaoning Normal University, No.1 Liushu South Street, Dalian, 650081 China; 2 State Key Laboratory of Biocontrol and Guangdong Provincial Key Laboratory of Plant Resources, School of Life Science, Sun Yat-Sen University, Guangzhou, 510275 China; 3 Dandong Forestry and Grassland Development Service Center, Dandong, 118000 China; 4 State Key Laboratory of Desert and Oasis Ecology, Xinjiang Institute of Ecology and Geography, Chinese Academy of Sciences, Urumqi, 830011 China

**Keywords:** Endophytic bacterial community, High throughput sequencing, Dimorphic seeds, *Suaeda glauca*

## Abstract

**Background:**

Seed dimorphism has been thought to be a bet-hedging strategy that helps plants survive in the disturbed environment and has been widely studied for its ecological adaptation mechanism. Many studies showed that seed-associated microorganisms play an important role in enhancing plant fitness, but information regarding endophytic bacteria associated with dimorphic seeds is limited. This study explores the influence of seed coat structure and seed phytochemical properties on the community composition and diversity of endophytic bacteria of dimorphic seeds of *Suaeda glauca*. In this study, we used 16S rRNA high-throughput gene sequencing method to compare the community composition and bacterial diversity between brown and black seeds of *Suaeda glauca*.

**Results:**

A significant difference was observed in seed coat structure and phytochemical properties between brown and black seeds of *S. glauca*. Total 9 phyla, 13 classes, 31 orders, 53 families, 102 genera were identified in the dimorphic seeds*.* The dominant phyla were *Proteobacteria*, *Firmicutes*, and *Actinobacteria.* The results showed that seed dimorphism had little impact on the diversity and richness of endophytic bacterial communities but significantly differs in the relative abundance of the bacterial community between brown and black seeds. At the phylum level, *Actinobacteria* tend to be enriched significantly in brown seeds. At the genus level, *Rhodococcus*, *Ralstonia*, *Pelomonas* and *Bradyrhizobium* tend to be enriched significantly in brown seeds, while *Marinilactibacillus* was mainly found in black seeds. Besides, brown seeds harbored a large number of bacteria with plant-growth-promoting traits, whereas black seeds presented bacteria with enzyme activities (i.e., pectinase, cellulolytic and xylanolytic activities).

**Conclusion:**

The endophytic bacterial community compositions were significantly different between dimorphic seeds of *Suaeda glauca*, and play an important role in the ecological adaptation of dimorphic seeds by performing different biological function roles. The endophytic bacterial communities of the dimorphic seeds may be influenced mainly by the seed coat structureand partly by the seed phytochemical characteristics. These findings provide valuable information for better understanding of the ecological adaptation strategy of dimorphic seeds in the disturbed environment.

**Supplementary Information:**

The online version contains supplementary material available at 10.1186/s12866-021-02206-1.

## Background

Seed dimorphism is thought to be a bet-hedging strategy where plant species produce two distinct types of seed within the same plant [1, 2], and usually associated with differences in seed size, shape, color and absence/presence of seed appendages [3–5]. Seed dimorphism is a common phenomenon in the halophyte such as *Suaeda* spp., observed in *S. glauca* [6], *S. salsa* [7–10], *S. acuminate* [11], *S. aralocaspica* [12, 13], *S. corniculata* sp. *mongolica* [14], *S. splendens* [2] and *S. moquinii* [15].

In the past few years, researchers have studied the ecological adaptation mechanism of *Suaeda* spp. related to seed dimorphism, which primarily focused on seed ecological behaviors including seed germination/dormancy traits [7, 11, 12, 15], competitive abilities [8] and phenotypic plasticity [10, 14]; seed physiological properties including seed coat structure [16] and seed phytochemical characteristics (ion content, nutrient composition) [16–18]; as well as transcriptome analysis of dimorphic seeds during germination [13]. However, with the emergence of the concept of the “holobiont” [19, 20], plants are no longer viewed as monogenetic individuals but as polygenetic entities, where the microbiota plays an important role in the ecological adaptation of plants [21]. Seed endophytic bacteria have been reported to influence seed germination [22, 23], seed preservation [24], seedling establishment and development [23, 25–27], as well as play an important role in enhancing plant fitness [28]. Besides enhancing plant fitness, they also help the plant to tolerate stress conditions [29].

Numerous studies have shown that the composition of seed endophytic microbiota not only influenced by soil factors [30] and plant genotype [31–33], but also by seed phytochemical traits (including antioxidants content, starch content and nutrition component) [34, 35] and seed physiological characteristics (such as salt tolerance) [33]. Interestingly, previous studies showed that the dimorphic seeds of *Suaeda* spp. exhibit significant differences in seed phytochemical properties including fatty acid composition [18], total unsaturated fatty acids content [36], total phenols, flavonoids, carotenoids content [17], soluble sugar, soluble protein, total nitrogen, total phosphorus, inorganic ion content [16], seed coat structure [16] and seed salt-tolerance [7, 9, 37–39]. So, we hypothesized that differential endophytic bacterial communities can be detected between two distinct types of seeds of *Suaeda* spp., and may perform different bacterial function roles.

*Suaeda glauca* Bunge, a common annual halophyte, produces two distinct types of fruit (large utricles vs small utricles) and exhibits different germination behavior [6]. The present study aims to explore as follows:

(1) observe the morphological structure difference between the dimorphic seeds of *S. glauca*, (2) investigate the difference in the seed phytochemical characteristics (soluble protein, soluble starch, soluble sugar, fat content, and total phenols) between the dimorphic seeds of *S. glauca*, (3) compare the difference in the composition of the endophytic bacterial communities between the two distinct seed types, (4) provide useful information to understand the ecological role of the seed-associated endophytic bacterial community and to understand the ecological adaptation strategy for dimorphic seeds.

## Results

### Seed morphology and phytochemical properties

**Fig. 1:**
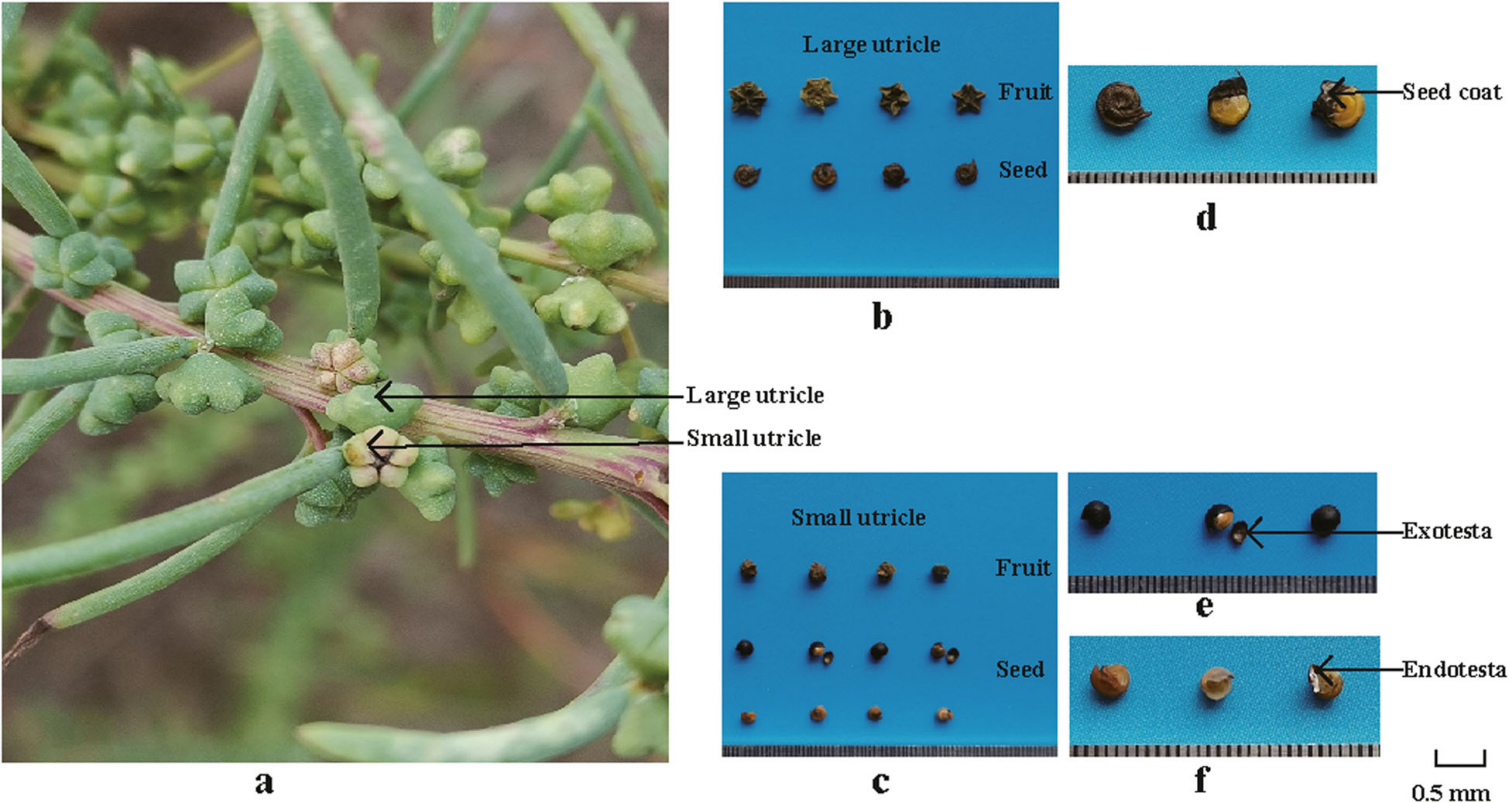
*Suaeda glauca*. **a** Positions of large utricles and small utricles of *S. glauca* on a branch in fruiting stage. **b** Fruit and seed morphological characteristics of large utricles in mature stage. **c** Fruit and seed morphological characteristics of small utricles in mature stage. **d** Morphological characteristics of seed coat of brown seed. **e** Morphological characteristics of exotesta of black seed. **f** Morphological characteristics of endotesta of black seed

The spatial site distribution pattern of dimorphic utricles (seeds) of *S. glauca* was observed in the mother plant grown in the same natural environment. Glomerules of *S. glauca*, usually inserted near the base of the leaves, usually consisted of 1–3 flowers. The glomerules at the top of the branches were one flower, which forms a large utricle, while the glomerules at the middle and lower axils of the branches usually consisted of three flowers, which produces three utricles. The two large utricles were usually located on the two lateral sides of the glomerules and the small utricles in the middle (Fig. 1a).

Indoor observations showed that *S. glauca* produced two types of utricles: large utricles, pentagram-shaped, with five expanded tepals in the fruiting stage, which surround and protect brown seeds (Fig. 1b); whereas small utricles, spheroid-shaped, with non-expanded tepals in the fruiting stage, which protect black seeds (Fig. 1c). At maturity, the brown seed had only a soft and membranous seed coat (Fig. 1d). On the contrary, the black seed had a rigid cuticle exotesta (Fig. 1e) and a soft membranous endotesta (Fig. 1f). The hard shell of the exotesta resists strong inward pressure at maturity. The results showed significant differences in fruit size, seed size, seed coat structure between brown and black seeds.

**Fig. 2:**
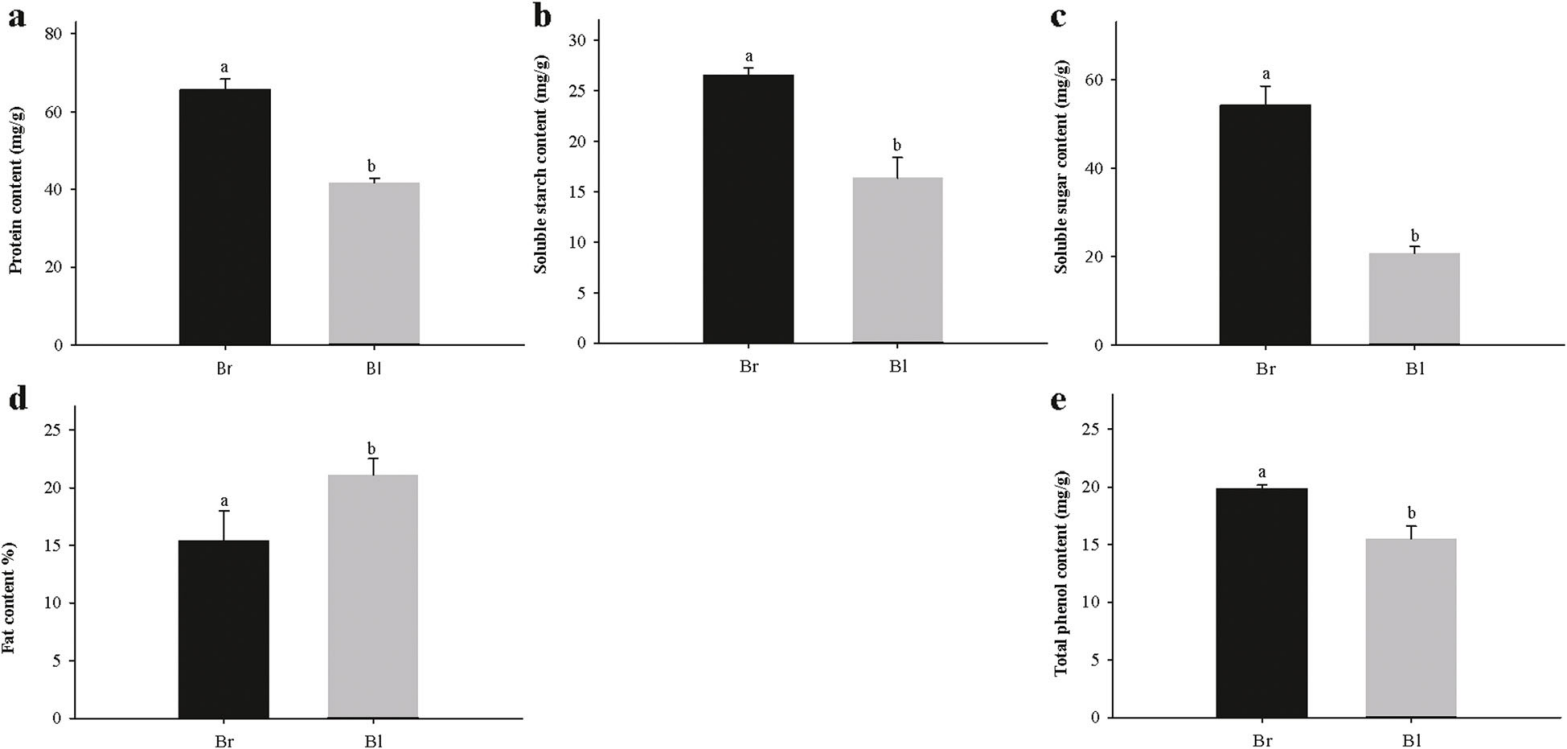
Comparison analysis of five seed phytochemical properties between brown seeds and black seeds. (a) Protein content; (b) Soluble starch content; (c) Soluble sugar content; (d) Fat content; (e) Total phenolic content. Results are presented as means of three replicates and vertical bars indicate standard deviations of the means. Different letters indicate significant differences between two types seed according to Student’s T-test at *p* < 0.05. Br: brown seeds; Bl: black seeds

The phytochemical properties of the seeds were tested and the results showed a very significant difference in protein, soluble starch, soluble sugar and total phenolic content between brown and black seeds. As shown in Fig. 2, protein, soluble starch, soluble sugar and total phenolic content in brown seeds were higher than those in black seeds. In contrast, the fat content of brown seeds was lower than that of black seeds.

### Characteristics of 16S rRNA gene sequencing and alpha-diversity

The surface-sterilized seeds placed on TSA agar plates showed no microbial growth (Additional file 1: Fig. S1). It was therefore assumed that the bacteria identified from all seed samples were endophytic, or very closely associated with seed epidermal tissue.

The bacterial 16S rRNA gene (V5-V6 regions) was sequenced to characterize the endophytic bacterial community composition between brown and black seeds. After the quality filtration of raw data, a total of 114,770 high-quality sequences were obtained from 6 samples. The mean sequence number per sample was 19,128 ± 5604, ranging from11,433 to 24,822 (Table 1) (each sample = 0.20 g seeds weight and each group = 3 samples). The sequence numbers, coverage, the number of operational taxonomic units (OTUs), richness, and diversity indices for each sample were presented in Table 1. The high-quality sequences were clustered into 175 OTUs (at 97% sequence identity) and each library contains different phylogenetic OTUs ranging from 29 to 120.

**Table 1:** Richness and diversity indexes in each sample (OUT cut off 0.03)

Sample ID	Sequence numbers	Average length (bp)	Coverage	Number of OTUs	Alpha diversity			
					ACE	Chao	Shannon	Simpson
Brown seeds								
Br_1	11,433	376.20	0.999238	120	121.687	112.5	2.5209	0.177828
Br_2	23,759	377.65	0.997334	48	98.71399	83	1.315757	0.373687
Br_3	23,206	375.00	0.999746	83	83.51684	83.33333	2.596081	0.15705
Black seeds								
Bl_1	14,223	376.83	0.997334	50	102.2935	80	1.316911	0.324566
Bl_2	17,327	377.25	0.997461	50	105.8463	65.83333	1.125242	0.408601
Bl_3	24,822	377.45	0.998096	29	55.23336	55.25	1.347446	0.33234

OTUs were defined at the 97% similarity level (threshold is 0.03)

*Br* Brown seeds, *Bl* Black seeds

**Fig. 3:**
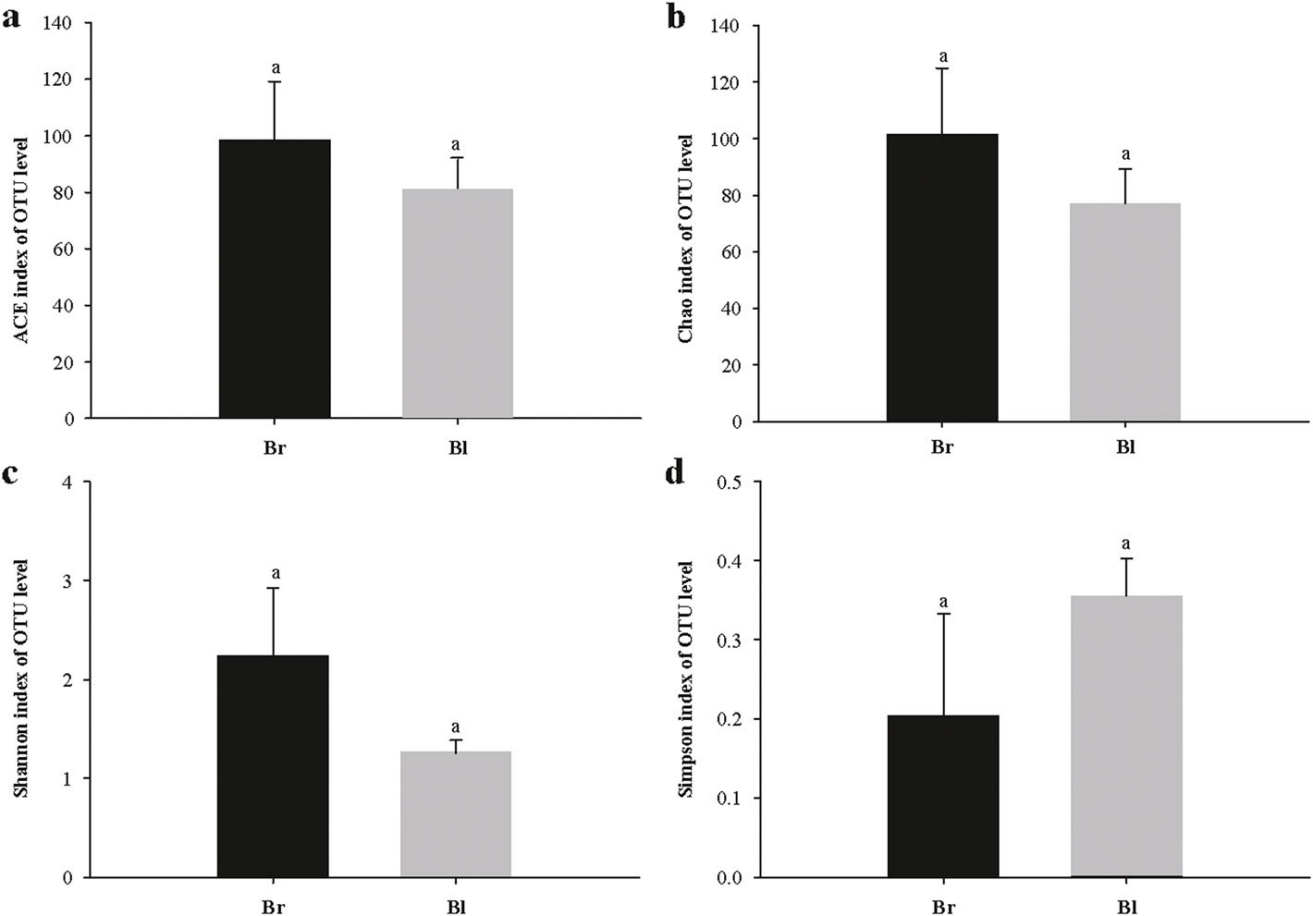
Comparison of the richness and diversities of bacterial OUT level between brown seeds and black seeds. (a) ACE index; (b) Chao index; (c) Shannon index; (d) Simpson index. Results are presented as means of three replicates and vertical bars indicate standard deviations of the means. Different letters indicate significant differences between two types seed according to Student’s T-test at *p* < 0.05. Br: brown seeds; Bl: black seeds

The diversity and richness indices of all samples were calculated to illustrate the complexity of each sample (Table 1). The diversity of each sample was obtained by using Shannon index and Simpson index. The Shannon index ranged from 1.125 to 2.596, while the Simpson index ranged from 0.157 to 0.409. The Chao index and ACE index were usually used to express the richness of each sample. Chao index ranged from 55.250 to 112.500, while ACE index ranged from 55.233 to 121.687. In total, the values of ACE, Chao, Simpson and Shannon varied among six samples. However, no significant differences in all alpha-diversity estimators were observed between brown and black seed populations (*p* > 0.05, student’s *t*-test; Fig. 3). The Good’s coverage value per sample was > 0.99 (from 0.997 to 0.999), indicating that the information was sufficient to reveal most of the bacterial communities in each sample.

### Taxonomic composition of endophytic bacterial community

**Fig. 4:**
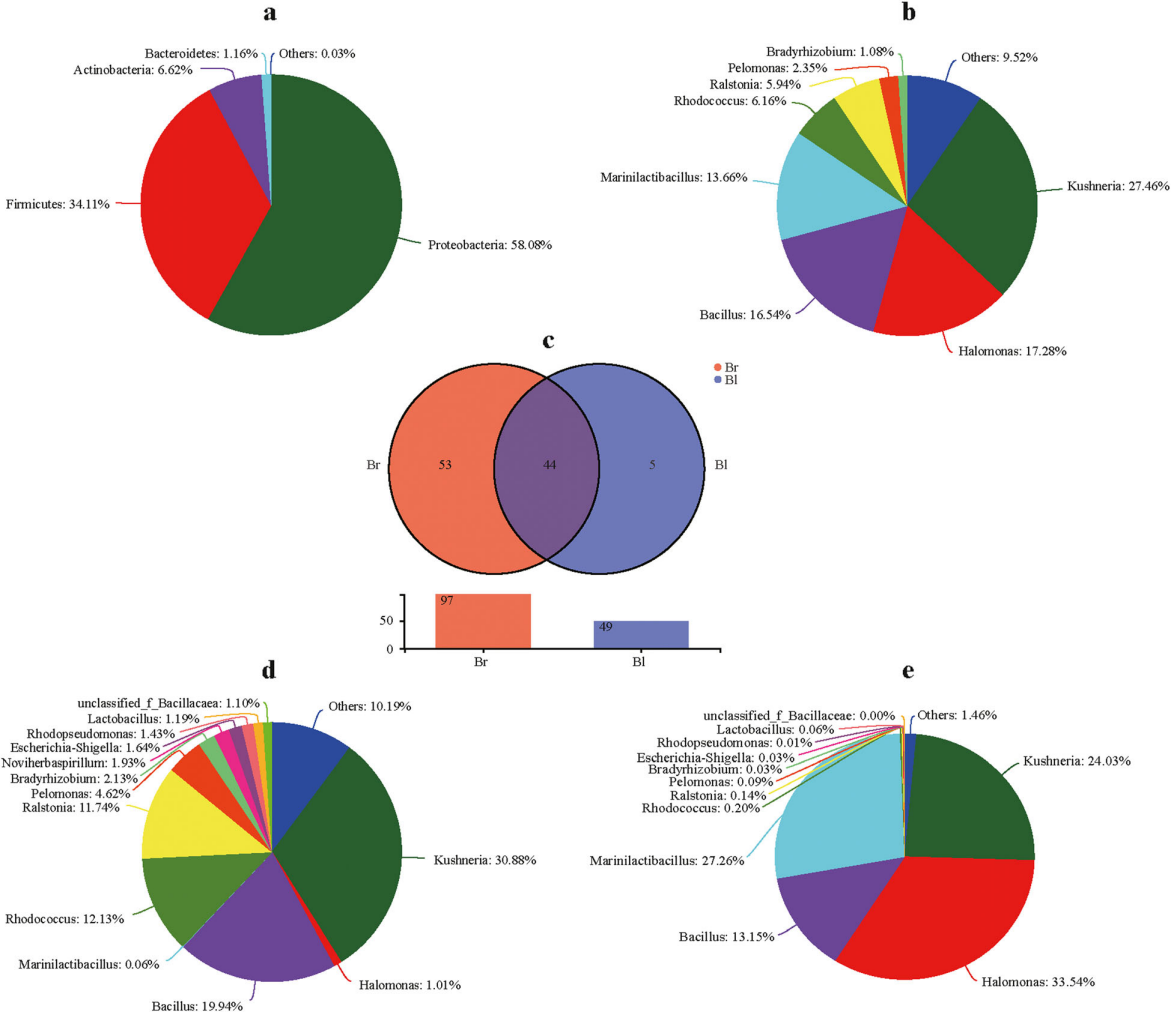
The bacterial community in all seed samples at phylum level (**a**), genus level (**b**). The comparison (**c**) of the endophytic bacterial communities at genus level between brown seeds and black seeds. The community composition of endophytic bacteria of brown seeds (**d**), and black seeds (**e**) at genus level, respectively. 3 samples in each group. Each sample = 0.20 g seeds

The 16S rRNA gene sequence results showed that the microbial communities of all seed samples covered 9 phyla, 13 classes, 31 orders, 53 families, 102 genera and 137 species. Sequences that were less than 1.0% in abundance were merged into “others”. The relative abundant phyla in all samples were *Proteobacteria* (58.0%), *Firmicutes* (34.1%), *Actinobacteria* (6.6%), *Bacteroidetes* (1.1%) and others (0.03%) (Fig. 4a).

There were 19 genera with a relative abundance of > 1.0% in at least one of the six samples (Additional file 3: Table S1). Of all 19 genera, 8 classified genera (average relative abundance more than 1.0% at genus level) were *Kushneria* (27.4%), *Halomonas* (17.2%), *Bacillus* (16.5%), *Marinilactibacillus* (13.6%), *Rhodococcus* (6.1%), *Ralstonia* (5.9%), *Pelomonas* (2.3%) and *Bradyrhizobium* (1.0%) (Fig. 4b). The Venn diagram (Fig. 4c) at the genus level was also constructed to further identify the shared genus present in brown and black seeds. The results suggest that 44 genera were shared between the two groups. The core genera present in the dimorphic seeds of *S. glauca* were *Kushneria* (24.0–30.8%), *Bacillus* (13.1–19.9%) and *Holomonas* (1.0–33.5%) (Fig. 4d and e). In addition, more bacterial taxa were found in brown seeds (53 genera), compared to black seeds (5 genera) (Fig. 4c).

### Community analysis of endophytic bacterial compositions

**Fig. 5:**
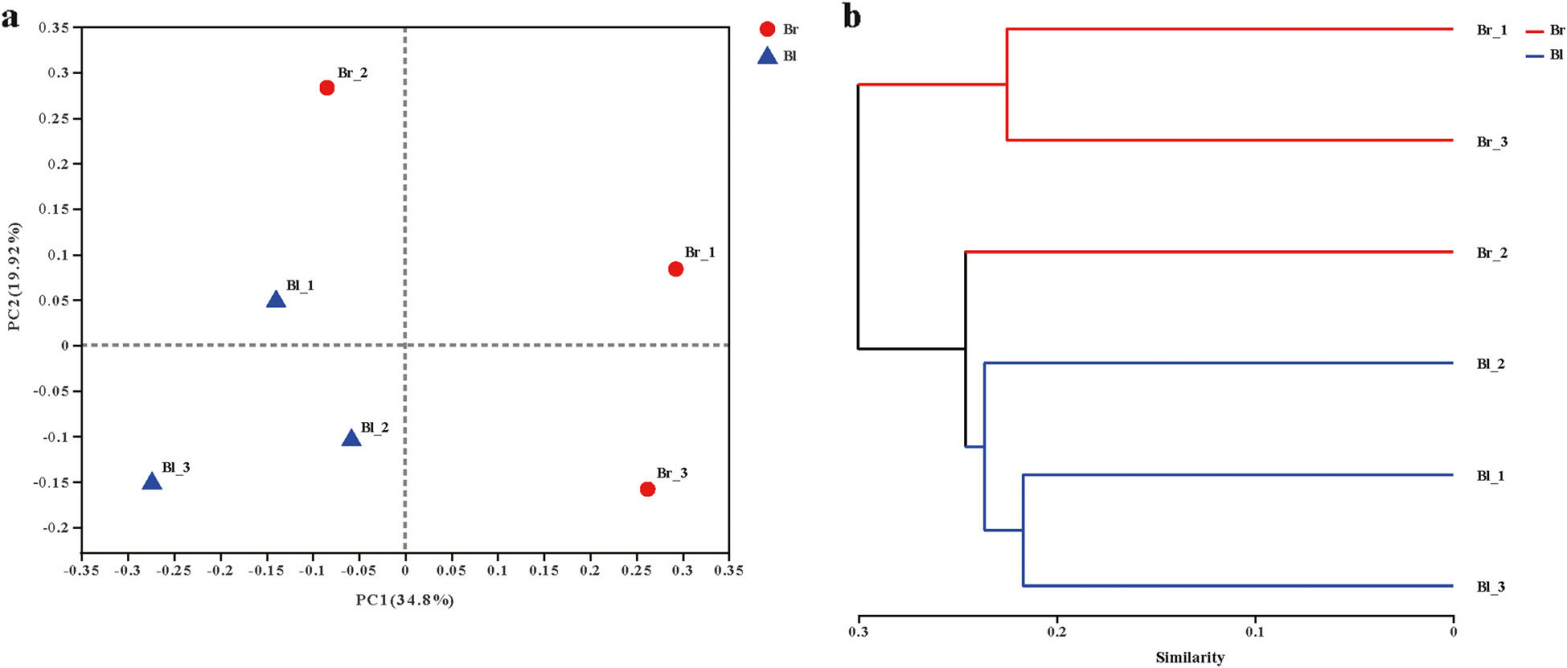
The principal co-ordinates analysis (PCoA) (**a**) and hierarchical clustering tree (**b**) of the bacterial community at OTU level in the two groups. 3 samples in each group. Each sample = 0.20 g seeds. The hierarchical clustering tree was calculated using the unweighted unifrac method, and the relationship between samples was determined by the complete clustering method

Principal coordinate analysis (PCoA) was used to determine the similarity of the endophytic bacterial communities between brown and black seeds. The PCoA biplot revealed that all seed samples show a clear separation across PC1 axis, except for Br_2 sample, which coincides with the different seed types (Fig. 5a). The results showed that all black seed samples were grouped in the left of the biplot and presented better similarity in their endophytic bacterial communities, however, there were considerable differences in endophytic bacterial communities of brown seeds. In total, black and brown seed samples were separated along the first axis (PC1), explaining 34.8% and the second axis (PC2) of explaining 19.9%.

In addition, the hierarchical clustering of the endophytic bacterial communities (OTUs level at 97% similarity) in both brown and black seeds was conducted based on the unweighted uni-fraction method (Fig. 5b), which reflects that the bacterial communities appeared different between the two seed types. The result of the hierarchical clustering was similar to that of PCoA analysis.

Interestingly, these results also revealed high heterogeneity within the bacterial communities associated with brown seeds. In contrast, the bacterial community composition of black seeds was very similar in all treatments. Hence, it was concluded that black seeds offer a more stable and less easily disturbed environment compared to brown seeds.

### Differences in the endophytic community compositions

**Fig. 6:**
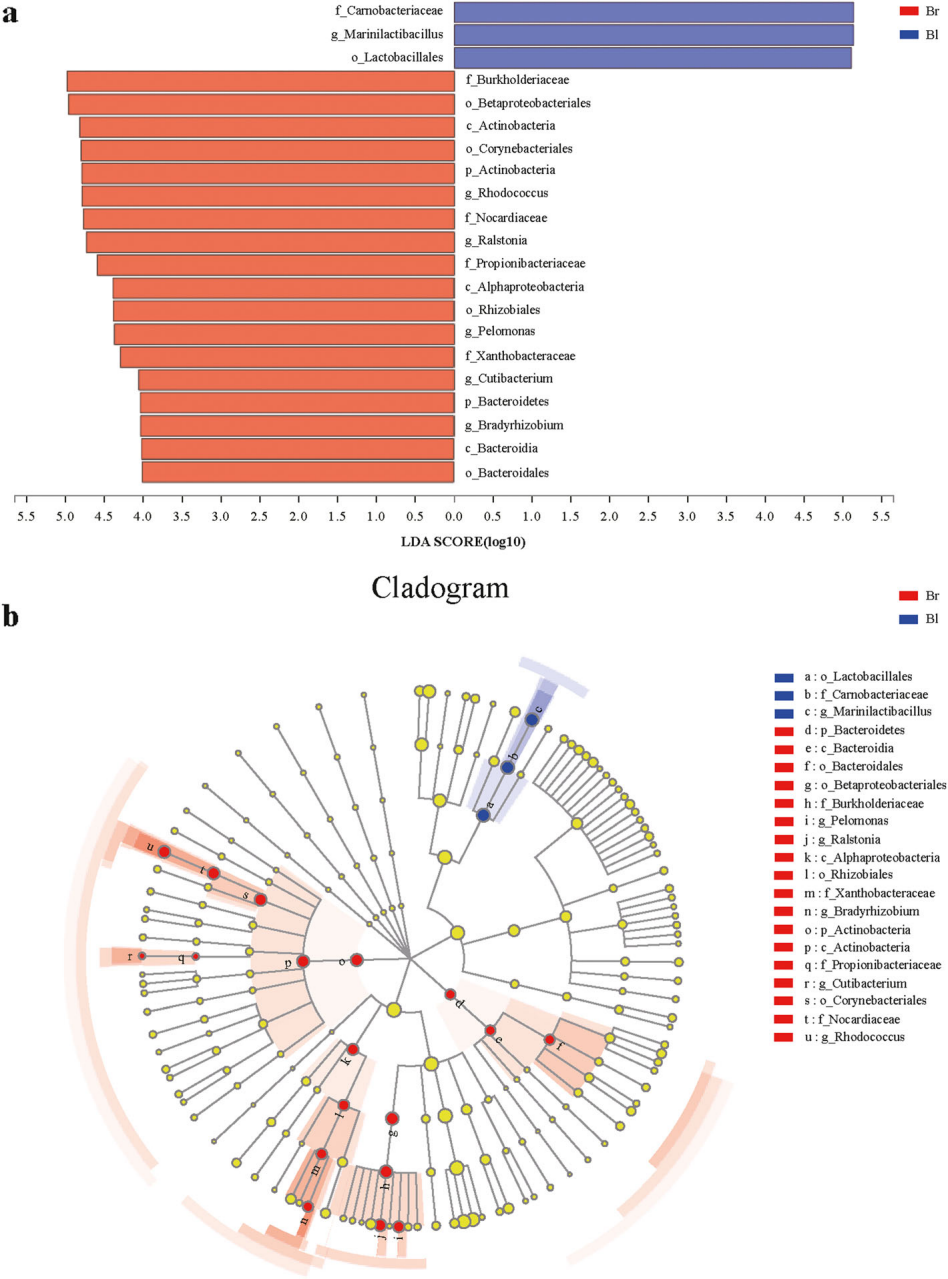
LEfSe analyses of bacterial community in the brown seeds and black seeds. Br: brown seeds; Bl: black seeds. **a** Histogram of the microbiota of brown seeds and black seeds with a threshold value of 4; *P* < 0.05 considered significant. **b** Cladogram representing the abundance of the taxa in the brown seeds and black seeds

Linear Discriminant Analysis Effect Size (LEfSe) analysis was used to determine the significant differences of the bacterial communities between brown and black seeds. Illumina MiSeq sequencing data demonstrated that the relative abundances of bacterial taxa have displayed significant differences between brown and black seeds at the phylum, class, order, family and genus level (Fig. 6).

At the phylum level, the dominant phyla (relative abundance > 5.0% at least in one sample) were *Proteobacteria* (Br = 57.4 ± 19.9% and Bl = 58.6 ± 22.8%), *Firmicutes* (Br = 27.3 ± 30.9% and Bl = 40.8 ± 22.5%) and *Actinobacteria* (Br = 13.0 ± 13.2% and Bl = 0.2 ± 0.1%). Of them, *Proteobacteria* was the most dominant phylum in both brown and black seeds. The differences in the relative abundance of *Actinobacteria* (LDA = 4.790, *p* = 0.0495) exhibited a significant difference between brown and black seeds. Compared with black seeds, *Actinobacteria* was significantly enriched in brown seeds. On the contrary, the relative abundance of *Firmicutes* in brown seeds was less than that of black seeds, but there was no significant difference between the two groups. Detailed the relative abundance information of endophytic bacteria at phylum level in brown seeds and black seeds can be found in the Additional file 4: Table S2.

In the observed 102 identified genera, the dominant genera (relative abundance > 5.0% at least in one sample) were *Kushneria* (Br = 30.8 ± 27.2% and Bl = 24.0 ± 15.1%), *Halomonas* (Br = 1.0 ± 0.9% and Bl = 33.5 ± 28.1%), *Bacillus* (Br = 19.9 ± 34.2% and Bl = 13.1 ± 22.7%), *Marinilactibacillus* (Br = 0.06 ± 0.1% and Bl = 27.2 ± 15.2%), *Rhodococcus* (Br = 12.1 ± 12.3% and Bl = 0.2 ± 0.16%), *Ralstonia* (Br = 11.7 ± 13.8% and Bl = 0.14 ± 0.16%), *Pelomonas* (Br = 4.6 ± 5.0% and Bl = 0.09 ± 0.03%) and *Bradyrhizobium* (Br = 2.1 ± 2.7% and Bl = 0.03 ± 0.01%). Of them, *Marinilactibacillus* (LDA = 5.134, *p* = 0.0495), *Rhodococcus* (LDA = 4.785, *p* = 0.0495), *Ralstonia* (LDA = 4.734, *p* = 0.0495), *Pelomonas* (LDA = 4.4.373, *p* = 0.0495) and *Bradyrhizobium* (LDA = 4.033, *p* = 0.0495) exhibited significantly differences between the two groups (Fig. 6a). The relative abundances of *Rhodococcus*, *Ralstonia*, *Pelomonas* and *Bradyrhizobium* were significantly enriched in brown seeds, while *Marinilactibacillus* was enriched in black seeds. Although, *Kushneria*, *Halomonas* and *Bacillus* haven’t exhibited significant differences between the two groups, but they presented different distribution proportions between the two groups. Detailed the relative abundance information of endophytic bacteria at genus level in brown seeds and black seeds can be found inthe Additional file 3: Table S1.

The relationship between two sample groups and dominant endophytic bacteria at species level could be found in Additional file 2:Fig. S2. The results showed that *Bacillus krulwichiae*, *Rhodococcus erythropolis*, *Ralstonia solanacearum*, *Pelomonas* (unclassified), and *Bradyrhizobium elkanii* had higher relative abundance in brown seeds than in the black seeds, whereas the black seeds harbored a high relative abundance of unclassified *Halomonas* (unclassified), *Marinilactibacillus* (unclassified) and *Bacillus gibsonii*.

The results showed that the relative abundance of bacterial community distribution patterns between brown and black seeds collected from the same natural environment differed significantly.

### Functional analysis of the microbiota

**Fig. 7:**
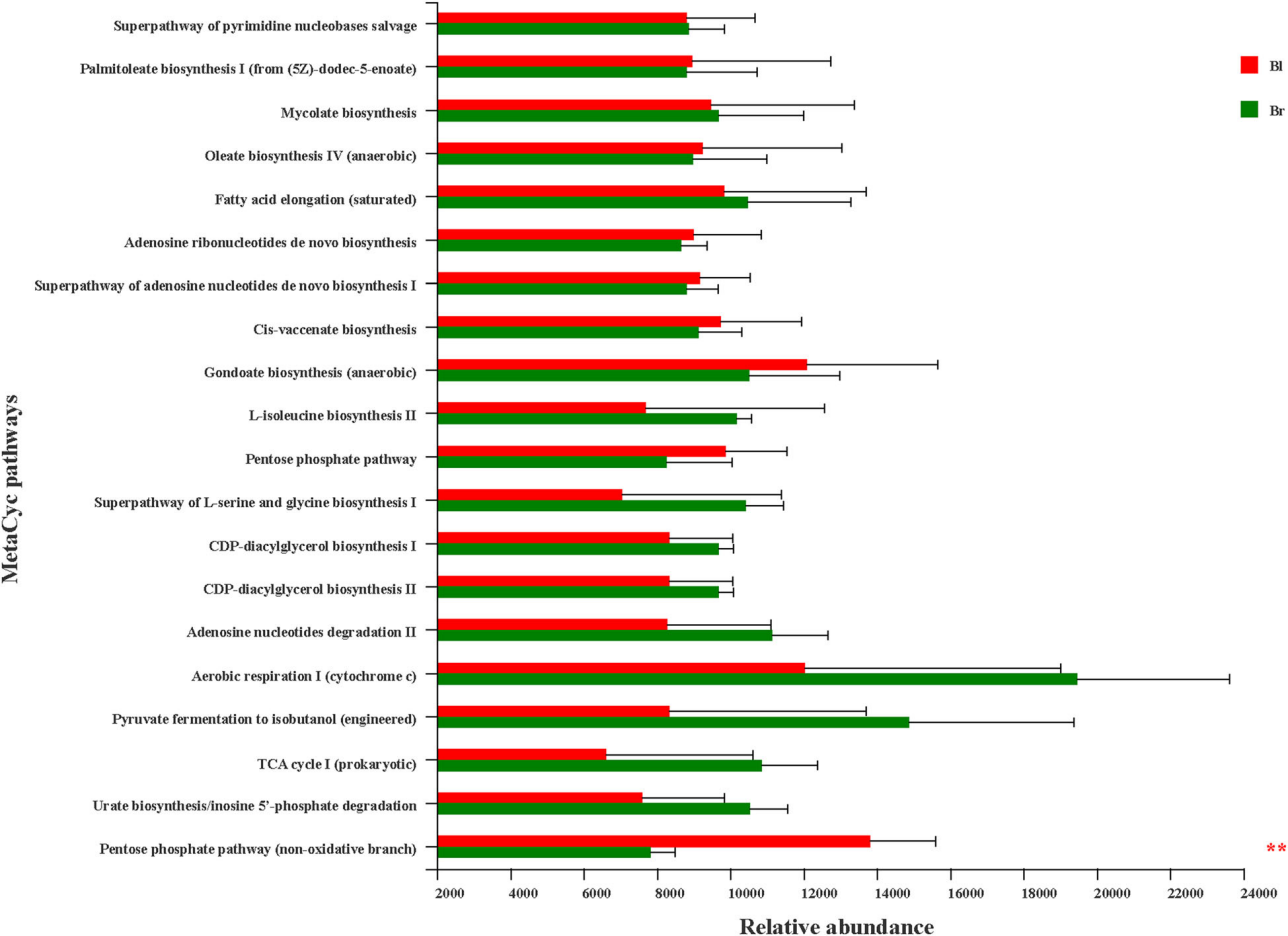
Comparison of the relative abundance in top 20 MetaCyc metabolic pathways between brown seeds and black seeds. Br: brown seeds; Bl: black seeds. * stands for 0.01 ≤ *p* < 0.05, ** stands for 0.001 ≤ *p* < 0.01 and *** stands for *p* < 0.001 according to Student’s T-test

The presumptive functions of the endophytic microbiota of the dimorphic seeds collected from the same natural environment were evaluated using PICRUSt2. The predicted genes were classified by aligning them to the MetaCyc databases (https://metacyc.org/). A total of 370 metabolic pathways were identified and were further selected to analyze significant differences between the two groups. Within the top 20 relative abundance categories, the abundance of only pentose phosphate pathway (non-oxidative branch) exhibited significantly difference between the two groups (Fig. 7), which significantly higher in black seeds than in brown seeds.

## Discussion

In this study, high-throughput sequencing technology was used to reveal the diversity of endophytic bacterial communities in the dimorphic seeds of *S. glauca* obtained from the same natural environment. Our findings demonstrated that seed dimorphism had little impact on the diversity and richness of endophytic bacterial communities in brown and black seeds, but significantly different relative abundances of the endophytic bacterial taxa were detected in brown and black seeds of *S. glauca*.

Many studies have shown that the seed dimorphism of *Suaeda* spp. usually associated with differences in seed shape, seed size, seed coat color [2, 6–8, 11, 12, 14, 15], seed coat structure [16], seed germinability [7, 11, 12, 15] and seed phytochemical properties [16–18]. In this study, we observed that seed coat structure and seed phytochemical properties of brown and black seeds of *S. glauca* significantly differed. Our results revealed that black seeds of *S. glauca* had two layers of the seed coat, including a layer of hard, cuticle exotesta and a layer of soft, membranous endotesta compared to single-layer membranous testa in brown seeds. A similar result has also been reported in *Borszczowia aralocaspica* (*S. aralocaspica*) [16]. Seed coat acts asa modulator between seed and environment and can regulate gaseous exchange and water imbibition [40, 41]. A previous study has indicated that black seeds of *S. glauca* had an intermediate physical dormancy and exhibited a low germination percentage, but it was water-permeable [6]. Brits et al. [42, 43] demonstrated that the intact hard testa may partially reduce oxygen diffusion to the embryo, contribute to hypoxic constraints. Brits and Manning [44] found the seeds of *Leucospermum cordifolium* have also two layers of seed coat (exotesta and endotesta), and exhibit water-permeable and oxygen-impermeable, which named as “anoxia PY (physical dormancy)”. Besides, Wang et al. [10] reported that the seed coat of black seeds of *S. salsa* contains a high content of waxes compared to brown seeds. These results implied that the difference in both structure and chemical composition of seed coat leads to differences in oxygen exchange capacity between black and brown seeds of *S. glauca*. The black seeds may rather have a limited capacity for gas exchange compared to brown seeds. Interestingly, Tegtmeier et al. [45] found that oxygen availability can influence colonization patterns of microbes in the gut microbiota.

Our results revealed that the content of soluble protein, soluble starch and soluble sugar was significantly higher in extracts obtained from brown seeds than those of black seeds; in contrast, the fat content of brown seeds was lower than that of black seeds. The different abilities of nutrition accumulation in dimorphic seeds have also been reported in *S. salsa* [17, 36] and *S. aralocaspica* [16, 18]. For example, Song et al. [16] found that the content of soluble sugar, soluble protein, total nitrogen, total phosphorus and inorganic ions (K^+^, Na^+^, K^+^/Na^+^) in brown seeds were significantly higher than those of black seeds in *S. aralocaspica*. In addition, we also detected higher content of total phenols in brown seeds compared to that of black seeds. A similar result was also reported in *S. salsa* [17]. Overall, these results suggest that there were significant differences in seed phytochemical properties between the dimorphic seeds of *S. glauca*. Interestingly, numerous studies have determined that the compositions of seed endophytic microbiota have been influenced the seed phytochemical traits [34, 35].

In the present study, alpha-diversity indices were used to evaluate the seed endophytic bacterial community richness and diversity. The results showed no significant differences in alpha-diversity indices between brown and black seeds. It was quite surprising that the significant differences in the seed coat structure and seed phytochemical characteristics between brown and black seeds had little impact on the diversity and richness of endophytic bacterial communities in the dimorphic seeds. A similar result has also been reported by Zhang et al. [46], who found five rice genotypes have little impact on the diversity and richness of endophytic bacteria.

In the present study, 9 prokaryotic phyla were observed, of which *Proteobacteria*, *Firmicutes* and *Actinobacteria* were dominant. These above-mentioned phyla have also been reported as dominant endophytes of other plant seeds [46, 47]. *Kushneria*, *Halomonas*, *Bacillus*, *Marinilactibacillus*, *Rhodococcus*, *Ralstonia*, *Pelomonas* and *Bradyrhizobium* were the high relative abundant genera, of them, *Kushneria*, *Halomonas* and *Bacillus* were the core endophytic bacterial community. Interestingly, *Kushneria*, *Halomonas* and *Bacillus* have also been reported as dominant endophytes from roots of halophytes, such as *Salicornia rubra*, *Sarcocornia utahensis* and *Allenrolfea occidentalis* [48]. Previous studies have revealed that *Kushneria* strains were isolated mostly from saline environments [49], endosphere of halophyte *Arthrocnemum macrostachyum* [50] and *Avicennia germinans* [51], phyllosphere of halophyte *Avicennia germinans* [52] and rhizosphere of halophyte *Saccharum spontaneum* [53]. Some members of the genus *Kushneria* reported having plant growth-promoting activities, including siderophore production, indolacetic acid (IAA) production, nitrogen fixation and phosphate solubilization [50, 54]. *Halomonas* and *Kushneria* are closely related and were grouped in the same genus in the past [52]. Many *Halomonas* sp. exhibit salt tolerance and can improve plant growth under salt stress conditions [48, 55–57]. *Bacillus* is common genera among the endosphere niche of diverse plants, where they play an important role in plant protection and growth stimulation [58, 59]. The results suggested that these core taxa may play an important role in the seed endosphere of halophyte *S. glauca*, and these taxa can assist the plant to resist stress environments. Besides, the Venn diagram revealed that greater taxa presented in brown seeds, and also had high heterogeneity within the bacterial communities compared to black seeds (Fig. 5). One possible explanation was that brown seed with a single layer membraneous seed coat and abundant nutrients could contribute to colonize microorganisms present in the carposphere of utricles, and easily susceptible to the carposphere environment. Recent studies have shown that seed bacterial endophytes may also originate from the phyllosphere, anthosphere and carposphere [26, 60].

Based on alpha diversity analysis, PCoA analysis, and hierarchical clustering tree results, seed dimorphism had no significant impact on diversity indices as a whole, it influenced significantly the relative abundance of endophytic bacterial taxa between brown and black seeds. Our comprehensive comparison revealed that the relative abundances of endophytic bacterial communities of dimorphic seeds were significantly different from each other at phylum, class, order, family and genus level. At the phylum level, we observed 9 phyla. Interestingly, the relative abundance of *Actinobacteria* was higher in brown seeds than in black seeds, which means *Actinobacteria* may be enriched in brown seeds. This might attribute to brown seeds with single-layer membranous seed coat and fast germinability were easily susceptible to soil-borne pathogens compared to black seeds, while *Actinobacteria* may protect brown seeds against pathogens and promotes plant growth [61, 62]. Gripenberg et al. [63] found that there was a potential trade-off between seed chemical and mechanical defense, polyphenols are one of the most common seed defenses, which are most likely to be present in large seeds with short seed dormancy and low investment in mechanical seed defense. Compared to black seeds with high investment in mechanical seed defense (two layers seed coat, including hard exotesta and soft, membranous endotesta), brown seeds had a high level of phenolic content. Hence, we speculated that a high abundance of *Actinobacteria*, combined with high levels of total phenols, can protect brown seeds from pathogens in the soil.

At the genus level, 5 genera of the 8 dominant genera possessed significant differences between brown and black seeds. *Rhodococcus*, *Ralstonia*, *Pelomonas*, *Bradyrhizobium* and *Marinilactibacillus* exhibited a significant difference between the two groups. Notably, we found that *Rhodococcus*, *Ralstonia*, *Pelomonas* and *Bradyrhizobium* tend to be enriched in brown seeds and present in high proportion compared to black seeds. Our results revealed that *Rhodococcus erythropolis*, *Ralstonia solanacearam*, *Pelomonas* (species unclassified) and *Bradyrhizobium elkanii*, were the dominant species in brown seeds (Fig. S2). *Rhodococcus* have been found living in close association with various plant parts, such as the rhizosphere [64], phyllosphere [65, 66] and endosphere [67–70]. *R. erythropolis* can colonize plant roots [70], and also prevent plant disease by degrading N-acyl-homoserine lactone signaling molecules [71]. Moreover, several members of the genus *Rhodococcus* also show plant growth-promoting activities, including ACC deaminase, IAA production, siderophore production and phosphate solubilization [72–75]. Some strains of the genus *Pelomonas* were detected in the endosphere of *Typha angustifolia* [76] and reported to fix nitrogen [77]. *Bradyrhizobium,* a genus of Gram-positive that was initially proposed as a group of slow-growing, alkaline-producing root nodule nitrogen-fixing bacteria [78]. *B. elkanii* isolated from the root nodules of *Acacia confusa*, exhibit the nitrogen-fixing ability and can enhance the growth and root development of *A. confuse* [79]. Numerous studies revealed that endophytic bacteria can improve plant fitness by enhancing nutrient mobilization, nitrogen fixation, phosphate solubilization and conferring resistant against pathogens [27, 80]. Thus, we speculated that brown seeds harbor a large number of microorganisms with plant growth-promoting (PGP) traits, which contribute to the establishment and development of seedlings of brown seeds. Since brown seeds without dormancy behavior were the main source of early spring seedling of *S. glauca* [6]. In addition, we also detected strains of *Ralstonia* in brown seeds, such as *R. solanacearam*, which is an important soil-borne plant pathogen [81]. Taken together, it seemed that brown seeds served not only as a host for diverse plant-probiotic bacterial strains but also for putative opportunistic pathogenic bacteria.

In our study, compared to the endophytic microbiota of brown seeds, we found that *Marinilactibacillus* tends to be enriched in black seeds, and had higher proportions. Remarkably, *Marinilactibacillus* has also firstly reported as one of the most abundant genera in the endosphere of halophyte *Halimione portulacoides* [82]. A previous study revealed that *Marinilacibacillus piezotolerans* was a facultatively anaerobic *lactobacillus* [83, 84]. The results implied that *Marinilactibacillus* may adapt to the inner hypoxia environment of black seeds, since two layers of the seed coat of black seeds prevent gas-exchange. Truyens et al. [30] found that selection of seed endophytes partly relies on bacterial properties, and only bacteria with competitive and adaptive colonization characteristics can inhabit the seeds [85]. We also found that pentose phosphate pathway (non-oxidative branch) tends to be enriched in black seeds and had higher proportions compared to brown seeds. Stincone et al. [86] reported that the non-oxidation branch of the pentose phosphate pathway (PPP) is critical to maintain redox balance under stress situations. Fidalgo et al. [82] found that *Marinilactibacillus* spp. isolates tested positive for cellulolytic, proteolytic and xylanolytic enzymatic activities. Strain *B. gibsonii* (Fig. S2) was also an enriched species in black seeds, which was an efficient alkaline pectinase producer [87]. Together, the results suggested that oxygen availability may affect the competitive capacity of bacteria in endophytic microbiota of black seeds, and selectively enriched strains might reduce the mechanical resistance of hard exotesta of black seeds, which contributed to enhance the germinability of black seeds. Mayer and Poljakoo-Mayber [88] found that one of the possible reasons for the loss of impermeability of seeds was the action of microbes.

## Conclusion

In summary, our work revealed that seeds characteristics might play an important role in the endophytic bacterial composition of the dimorphic seeds of *S. glauca* collected from the same natural environment. The present study suggests that there were significant differences in seed coat structure and seed phytochemical properties between brown and black seeds of *S. glauca*. Although seed dimorphism had little impact on the diversity and richness of endophytic bacteria communities in brown and black seeds, a significant difference in the relative abundance of endophytic bacteria was detected. This study showed that under the same natural environmental conditions, the endophytic bacterial communities of the dimorphic seeds might be influenced mainly by the seed coat structure and partly by seed phytochemical characteristics. Moreover, this study also showed that seed fitness was closely associated with the variations of endophytic bacterial communities between brown and black seeds. Brown seeds harbored a large number of bacteria with plant-growth-promoting traits, whereas black seeds presented bacteria with enzyme activities (i.e. pectinase, cellulolytic and xylanolytic activities). These findings might provide valuable information for a better understanding of the ecological adaptation strategy of dimorphic seeds.

## Methods

### Seed collection and surface sterilization

The wild, naturally growing halophyte *S. glauca* were obtained from their natural habitats in yingchengzi coastal saline beach (121.36^°^ E, 38.99^°^ N) in Dalian, Liaoning, China. Mature seeds from naturally grown plants that colonized in the same natural environment were harvested (at least 100 mother plants collected on October 25th,2018) and air-dried for 10 days at room temperature.

The dimorphic seedswere separated according to their phenotypic characteristics, and then two types of seeds were placed into 50 ml sterile conical tubes. Each seed sample type was replicated three times. To avoid environmental bacterial contamination, seed surface sterilization was done according to the following procedure: First, the seeds were rinsed with 30 ml sterilized distilled water at least 5 times or until no cloudiness was observed in the wash. Second, the washed seeds were immersed in 1.0% sodium hypochlorite for 2 min. Third, the bleached seeds were rinsed with 30 ml sterile distilled water for 1 min and then immersed in 30 ml 70% ethanol for 1 min. Fourth, the ethanol was removed and seeds were rinsed five times with sterilized distilled water. Finally, the surface-sterilized seeds were air-dried for 12 h in the sterilized 90 mm Petri-dish with double filter paper. To check the effect of surface sterilization, some seeds per treatment were randomly picked and placed on the TSA agar medium (TSA, Qingdao Hope Bio-Technology Co., Ltd., Qingdao, P.R. China). The plates were incubated for 3 days at 25 °C. The sterilized seed samples were put in 50 ml sterile conical tube, frozen in liquid nitrogen and then immediately stored at − 80 °C for later DNA extraction.

### Seed morphological structure and phytochemical properties

The seed morphological structure was observed under the dissecting microscope. The crude fat, soluble sugar, soluble starch, total protein, total phenolic content was measured. To analyze the crude fat content, dry samples of dimorphic seeds (brown and black seeds, 1.0 g, respectively) were ground and petroleum ether (boiling range: 30 to 60 °C) was used as an extraction buffer. The crude fat extraction was performed using the Soxhlet apparatus. The crude fat content was determined following the AOAC method [89]. The procedure as described by Booij et al. [90] was followed for the extraction of soluble sugar with slight modification. 0.5 g dry seeds were crushed in a mortar using liquid nitrogen. Four milliliters of 80% ethanol was added and the mixture was incubated for 30 min at 100 °C.The extracts were centrifuged at 7000×g for 3 min at 4 °C. The supernatants were obtained and the extraction repeated twice. Soluble sugars were determined by the anthrone method at 625 nm using glucose as standard [91]. To analyze starch content, 0.5 g dry seeds were ground in a mortar using liquid nitrogen. The extraction was performed according to the method as described by Zhao et al. [92]. Starch content was determined with the anthrone method [93] at 640 nm using soluble starch as standard. For protein analysis, 0.5 g of dry seeds were ground and extracted according to the method as described by Piattoni et al. [94]. The total protein content was measured by using a spectrophotometer at 595 nm following the Bradford protocol [95]. Bovine Serum Albumin (BSA) standard curve was used to determine the total protein content. To analyze total phenolic content, 1.0 g dry seeds were ground and extracted according to the protocol described by Gallagher et al. [96]. Total phenolic content was determined using a spectrophotometer at 765 nm according to the Folin-Cocalteau reagent method [97]. Total phenolic concentrations were quantified by comparison with gallic acid as a standard curve.

### DNA extraction, PCR amplification and sequencing

Total genomic DNA was extracted from six surface-sterilized seed samples (0.2 g/each sample) using the E.Z.N.A.® soil DNA Kit (Omega Bio-Tek, Norcross, GA, U.S.) according to the manufacturer’s protocols. The quality of the genomic DNA was checked using 1% agarose gel electrophoresis. DNA concentration and purity were determined by NanoDrop 2000 UV-visible spectrophotometer (Thermo Scientific, Wilmington, USA).

Two-step PCR amplification was performed to minimize the host rRNA gene contamination while analyzing microbial communities. The first PCR amplification of the 16S rRNA gene was carried out with the bacterial primer pairs 799F (5′-AACMGGATTAGATACCCKG-3′) and 1392R (5′-ACGGGCGGTGTGTRC-3′) [98] on the ABI GeneAmp®9700 PCR thermocycler (ABI, CA, USA). The 20 μl PCR reaction mixture contained 10 ng template DNA, 5 × *TransStart* FastPfu buffer 4 μL, 2.5 mM dNTPs 2 μL, forward primer (5 μM) 0.8 μL, reverse primer (5 μM) 0.8 μL, *TransStart* FastPfu DNA Polymerase 0.4 μL, BSA 0.2 μL and finally ddH_2_O up to 20 μL. The PCR amplification of 16S rRNA gene was performed as follows: initial denaturation at 95 °C for 3 min, followed by 27 cycles of denaturing at 95 °C for 30 s, annealing at 55 °C for 30 s and extension at 72 °C for 45 s, and single extension at 72 °C for 10 min, and end at 4 °C. The second PCR amplification was performed using 2 μL of the extraction product as a template, using the bacterial 16S rRNA gene primer pairs 799F (5′-AACMGGATTAGATACCCKG-3′) and 1193R (5′-ACGTCATCCCCACCTTCC-3′) [99]. All conditions for the second PCR step were identical except that thermocycling was done for 13 cycles instead of 27 cycles. PCR reactions were performed in triplicate. The PCR product was checked by using 2% agarose gel electrophoresis, and purified using the AxyPrep DNA Gel Extraction Kit (Axygen Biosciences, Union City, CA, USA) according to manufacturer’s instructions and quantified using Quantus™ Fluorometer (Promega, USA).

Purified PCR products were sequenced by paired-end sequencing performed on an Illumina MiSeq PE300 platform (Illumina, San Diego, USA) according to the standard protocols by Majorbio Bio-Pharm Technology Co., Ltd. (Shanghai, China).

### 16S rRNA gene sequence analysis

The raw 16S rRNA gene sequences reads were demultiplexed, quality-filtered by fastp (version 0.20.0) [100] and merged by FLASH (version 1.2.7) [101] with the following criteria: (i) the 300 bp reads were truncated at any site receiving an average quality score of < 20 over a 50 bp sliding window, and the truncated reads shorter than 50 bp were discarded, reads containing ambiguous characters were also discarded; (ii) only overlapping sequences longer than 10 bp were assembled. The maximum mismatch ratio of the overlap region was 0.2. Reads that could not be assembled were discarded; (iii) samples were distinguished according to the barcode and primers. Chimeric sequences were identified and removed using UCHIME [102]. The remaining high-quality sequences were clustered into operational taxonomic units (OTUs) with 97% similarity cut-off [103, 104] using UPARSE (version 7.0) [104]. The taxonomy of each OTU representative sequence was analyzed by RDP Classifier (version 2.2) [105] against the SILVA 16S rRNA database (Release 132) using a confidence threshold of 0.7.

### Statistical analysis

To avoid biases introduced by primers, sequences belonging to chloroplasts (o_Chloroplast) or mitochondria (f_Mitochondria) were discarded and other OTUs of the libraries were used for microbial community analyses. Alpha-diversity was evaluated by calculating community richness parameters (Chao, ACE), community diversity parameters (Shannon, Simpson) and a sequencing depth index (Good’s coverage) using MOTHUR software (version v. 1.30.1) [106]. R package software (version 3.3.1) was used to generate the results of the Venn diagram, Bar diagram, Pie diagram and Circos diagram. Beta diversity analysis based on unweighted Unifrac was carried out to visualize the results of PCoA (Principal coordinates’ analysis) and hierarchical clustering analysis at the OUT level by using R package software. Besides, the prediction of the microbial gene functions was done using PICRUSt2 against the MetaCyc metabolic pathway database (https://metacyc.org/).

The student’s t-test (SPSS 19.0) was used to compare the difference of seed phytochemical properties, the Alpha-diversity index and the relative abundance of MetaCyc metabolic pathways between brown and black seeds. The differential bacterial taxa between brown and black seeds were analyzed using Linear discriminant analysis (LDA) effect size (LEfSe). Only taxa with an average relative abundance greater than 0.01% were considered. All reported values were the average of triplicate results (mean ± SD).

## Supplementary Information

**Additional file 1: Fig. S1.** Representative image of sterilized-surface dimorphic seeds on TSA agar medium incubated for 3 d at 25 °C. Br:brown seeds; Bl:black seeds.

**Additional file 2: Fig. S2.** The relationship between two sample groups and dominant endophytic bacterium at the species level. Br:brown seeds; Bl:black seeds.

**Additional file 3: Table S1.** The relative abundance of the genus in each sample (cutoff of 0.01).

**Additional file 4: Table S2.** The relative abundance of the phylum in each sample (cutoff of 0.01).

## Data Availability

The raw reads were deposited into the NCBI Sequence Read Archive (SRA) database (SRA, https://www.ncbi.nlm.nih.gov/sra) under accession number PRJNA664311 (https://dataview.ncbi.nlm.nih.gov/object/PRJNA664311?reviewer=nmr0cvtfvjbkm1lllc2tag9u5m). Other datasets used and/or analyzed during the current study are available from the corresponding author upon reasonable request.

## Abbreviations

TSA: Tryptone soy agar
PCoA: Principal co-ordinates analysis
OTUs: Operational taxonomic units
LEfSe: Linear discriminant analysis effect size
LDA: Linear discriminant analysis
PICRUSt: Phylogenetic Investigation of Communities by Reconstruction of Unobserved States
BSA: Bovine serum albumin
PCR: Polymerase chain reaction
Br: Brown seeds
Bl: Black seeds
