# Supplementary figures and images for: Insights into the endophytic bacterial community comparison and their potential role in the dimorphic seeds of halophyte Suaeda glauca
Source: BMC Microbiol. 2021 May 12;21:143. doi: 10.1186/s12866-021-02206-1 (PMC8114534; doi:10.1186/s12866-021-02206-1)

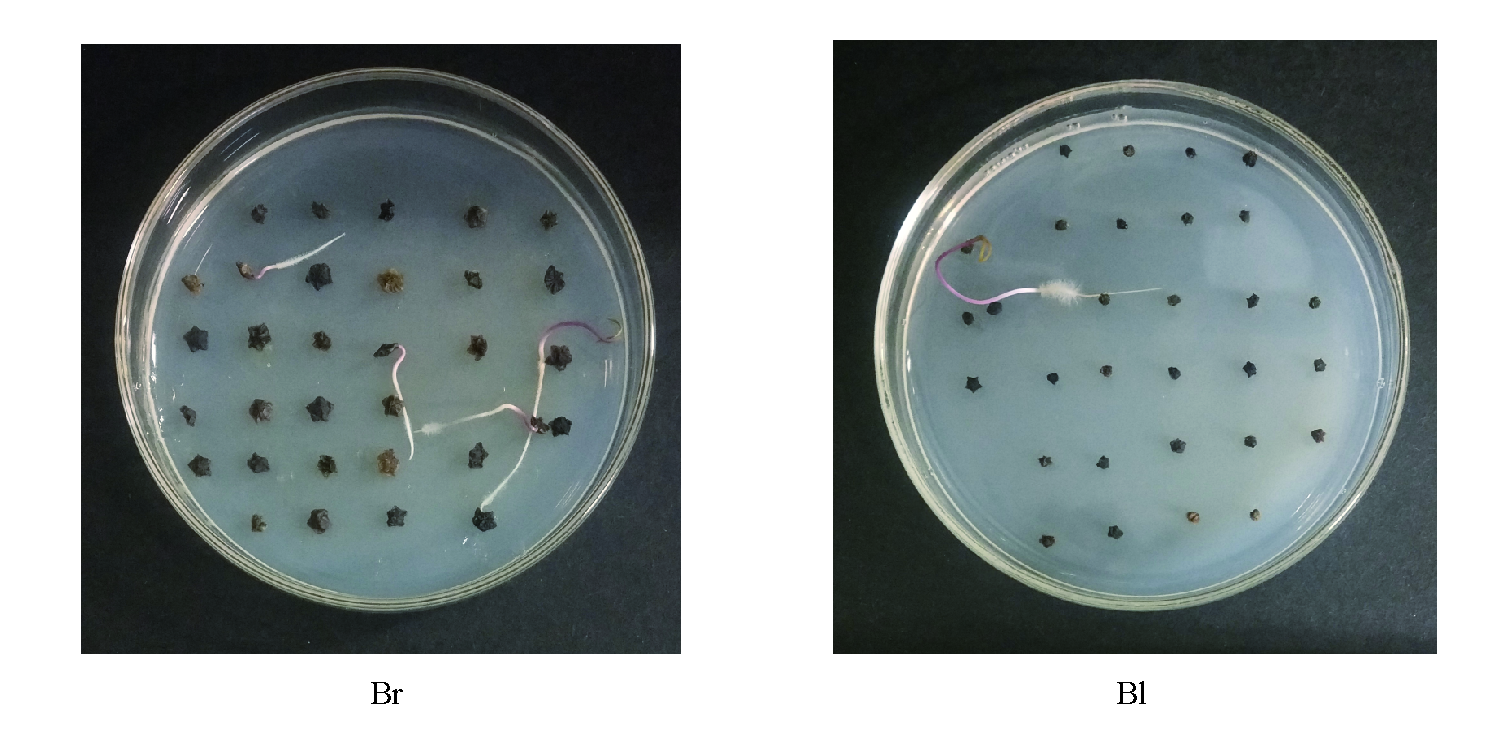

Supplement: Supplementary file 1 — Additional file 1: Fig. S1. Representative image of sterilized-surface dimorphic seeds on TSA agar medium incubated for 3 d at 25 °C. Br:brown seeds; Bl:black seeds. [file 12866_2021_2206_MOESM1_ESM.tif]

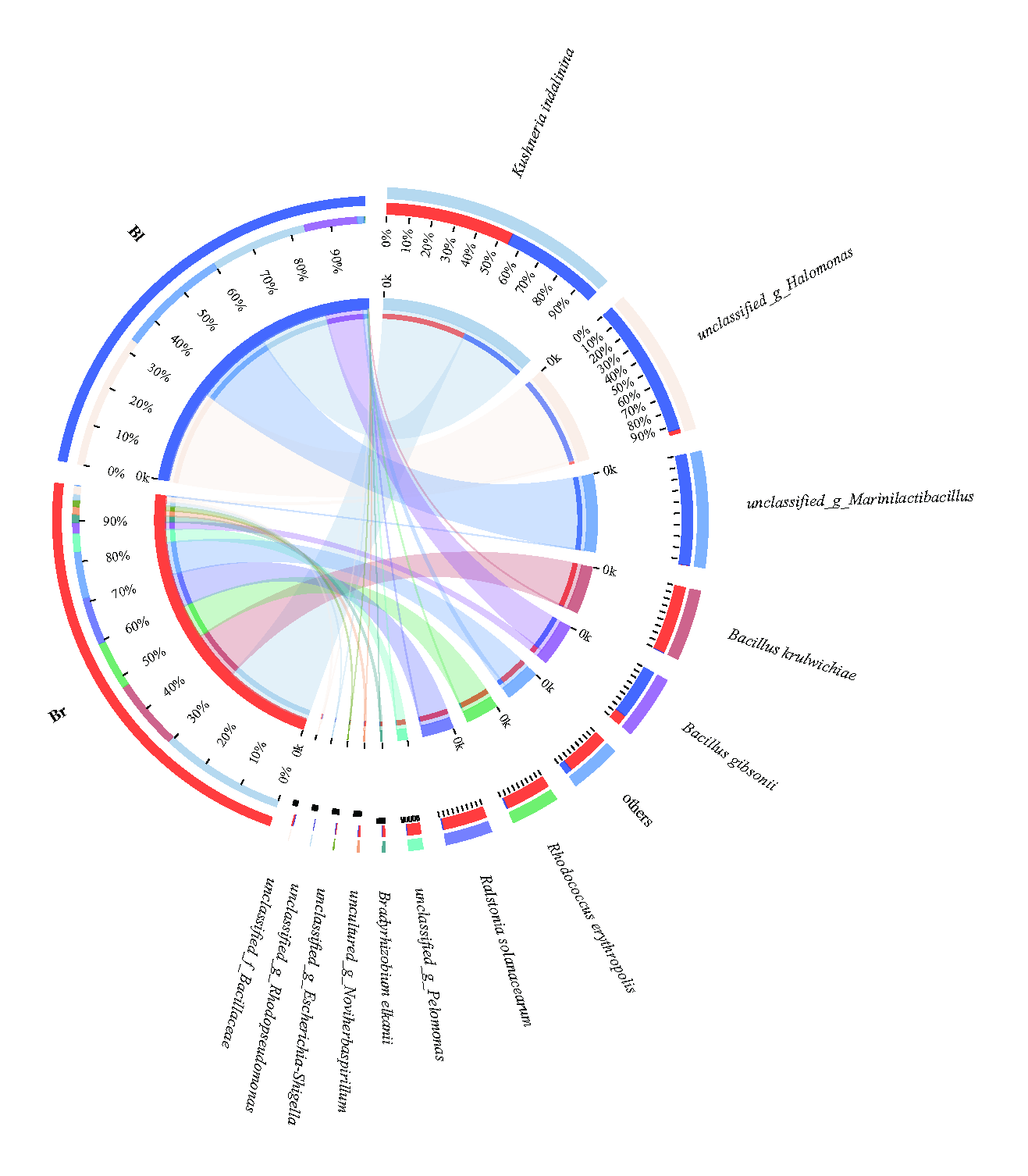

Supplement: Supplementary file 2 — Additional file 2: Fig. S2. The relationship between two sample groups and dominant endophytic bacterium at the species level. Br:brown seeds; Bl:black seeds. [file 12866_2021_2206_MOESM2_ESM.tif]
