# Supplementary material for: Insights into the endophytic bacterial community comparison and their potential role in the dimorphic seeds of halophyte Suaeda glauca
Source: BMC Microbiol. 2021 May 12;21:143. doi: 10.1186/s12866-021-02206-1 (PMC8114534; doi:10.1186/s12866-021-02206-1)
Supplement: Supplementary file 3 — Additional file 3: Table S1. The relative abundance of the genus in each sample (cutoff of 0.01). [file 12866_2021_2206_MOESM3_ESM.docx]

**Table S1. The relative abundance of the Genus in each sample (cutoff of 0.01).**

| Genus | Br_1 | Br_2 | Br_3 | Bl_1 | Bl_2 | Bl_3 |
| --- | --- | --- | --- | --- | --- | --- |
| *Kushneria*  *Halomonas*  *Bacillus*  *Marinilactibacillus*  *Rhodococcus*  *Ralstonia*  *Pelomonas*  *Bradyrhizobium*  *Escherichia-Shigella*  *Rhodopseudomonas*  *Lactobacillus*  *Noviherbaspirillum*  unclassified*_*f*_Bacillaceae*  *Lachnospiraceae*_nk4a136_group  *Phyllobacterium*  Unclassified*Lachnospiraceae*  *Salinicola*  *Staphylococcus*  *Prevotella_*9  others | 56.54%  1.1%  0.1%  0.01%  10.38%  6.63%  3.48%  1.07%  1.95%  1.17%  1.54%  1.5%  0.01%  1.28%  0.43%  0.29%  0.04%  1.12%  1.65%  9.71% | 33.76%  0.04%  59.52%  ND  0.71%  1.14%  0.25%  0.05%  0.09%  0.06%  ND  0.17%  3.27%  0.05%  0.01%  ND  ND  0.04%  0.22%  0.61% | 2.34%  1.89%  0.20%  0.18%  25.29%  27.46%  10.13%  5.28%  2.88%  3.07%  2.04%  4.13%  ND  1.57%  2.42%  1.49%  ND  ND  0.32%  9.32% | 35.96%  1.41%  39.44%  20.83%  0.38%  0.32%  0.1%  0.04%  0.04%  0.04%  0.08%  ND  0.01%  0.17%  0.01%  0.04%  0.38%  ND  ND  0.76% | 6.94%  45.56%  ND  44.69%  0.15%  0.11%  0.11%  0.03%  0.01%  ND  0.08%  ND  ND  0.13%  ND  0.15%  1.14%  0.01%  0.01%  0.86% | 29.2%  53.66%  ND  16.26%  0.06%  ND  0.05%  0.01%  0.03%  ND  0.03%  ND  ND  0.04%  ND  ND  0.37%  ND  ND  0.3% |

Note: Br: brown seeds; Bl: black seeds.
