# Supplementary material for: Insights into the endophytic bacterial community comparison and their potential role in the dimorphic seeds of halophyte Suaeda glauca
Source: BMC Microbiol. 2021 May 12;21:143. doi: 10.1186/s12866-021-02206-1 (PMC8114534; doi:10.1186/s12866-021-02206-1)
Supplement: Supplementary file 4 — Additional file 4: Table S2. The relative abundance of the phylum in each sample (cutoff of 0.01). [file 12866_2021_2206_MOESM4_ESM.docx]

**Table S2. The relative abundance of the phylum in each sample (cutoff of 0.01).**

| Phylum | Br_1 | Br_2 | Br_3 | Bl_1 | Bl_2 | Bl_3 |
| --- | --- | --- | --- | --- | --- | --- |
| *Proteobacteria*  *Firmicutes*  *Actinobacteria*  *Bacteroidetes*  others | 74.93%  9.44%  11.17%  4.34%  0.11% | 35.67%  63.02%  0.76%  0.55%  ND | 61.38%  9.55%  27.13%  1.46%  0.04% | 38.61%  60.83%  0.38%  0.18%  ND | 53.94%  45.44%  0.19%  0.41%  0.03% | 83.47%  16.39%  0.09%  0.05%  ND |

**Note**: Br: brown seeds; Bl: black seeds.
